# Investigation of Anticipatory Postural Adjustments during One-Leg Stance Using Inertial Sensors: Evidence from Subjects with Parkinsonism

**DOI:** 10.3389/fneur.2017.00361

**Published:** 2017-07-25

**Authors:** Gianluca Bonora, Martina Mancini, Ilaria Carpinella, Lorenzo Chiari, Maurizio Ferrarin, John G. Nutt, Fay B. Horak

**Affiliations:** ^1^Biomedical Technology Department, IRCCS Foundation Don Gnocchi Onlus, Milan, Italy; ^2^Department of Neurology, Oregon Health & Science University, Portland, OR, United States; ^3^Department of Electrical, Electronic and Information Engineering “Guglielmo Marconi”, University of Bologna, Bologna, Italy; ^4^VA Portland Healthcare Systems, VAPORHCS, Portland, OR, United States

**Keywords:** Parkinson’s disease, frontal gait disorders, anticipatory postural adjustments, wearable sensors, balance control, unipedal balance, single-leg stance

## Abstract

The One-Leg Stance (OLS) test is a widely adopted tool for the clinical assessment of balance in the elderly and in subjects with neurological disorders. It was previously showed that the ability to control anticipatory postural adjustments (APAs) prior to lifting one leg is significantly impaired by idiopathic Parkinson’s disease (iPD). However, it is not known how APAs are affected by other types of parkinsonism, such as frontal gait disorders (FGD). In this study, an instrumented OLS test based on wearable inertial sensors is proposed to investigate both the initial anticipatory phase and the subsequent unipedal balance. The sensitivity and the validity of the test have been evaluated. Twenty-five subjects with iPD presenting freezing of gait (FOG), 33 with iPD without FOG, 13 with FGD, and 32 healthy elderly controls were recruited. All subjects wore three inertial sensors positioned on the posterior trunk (L4–L5), and on the left and right frontal face of the tibias. Participants were asked to lift a foot and stand on a single leg as long as possible with eyes open, as proposed by the mini-BESTest. Temporal parameters and trunk acceleration were extracted from sensors and compared among groups. The results showed that, regarding the anticipatory phase, the peak of mediolateral trunk acceleration was significantly reduced compared to healthy controls (*p* < 0.05) in subjects with iPD with and without FOG, but not in FGD group (*p* = 0.151). Regarding the balance phase duration, a significant shortening was found in the three parkinsonian groups compared to controls (*p* < 0.001). Moreover, balance was significantly longer (*p* < 0.001) in iPD subjects without FOG compared to subjects with FGD and iPD subjects presenting FOG. Strong correlations between balance duration extracted by sensors and clinical mini-BESTest scores were found (ρ > 0.74), demonstrating the method’s validity. Our findings support the validity of the proposed method for assessing the OLS test and its sensitivity in distinguishing among the tested groups. The instrumented test discriminated between healthy controls and people with parkinsonism and among the three groups with parkinsonism. The objective characterization of the initial anticipatory phase represents an interesting improvement compared to most clinical OLS tests.

## Introduction

Ability to control anticipatory postural adjustments (APAs) prior to lifting one leg while standing in unsupported equilibrium represents a complex motor task that is significantly impaired by idiopathic Parkinson’s disease (iPD) (1, 2). Two types of parkinsonism, such as iPD and frontal gait disorders (FGD, also called lower body parkinsonism or vascular parkinsonism), result in similar tendencies to freeze with gait initiation, to walk with short, shuffling steps, and to fall frequently (3–7). However, it is not known how FGD affects APAs. The effects of different types of parkinsonism on APAs may differ because people with iPD stand and walk with a narrow base of support whereas people with FGD stand and walk with wider than normal base of support (8, 9). APAs prior to voluntary movement are known to improve with improvements in bradykinesia from levodopa replacement therapy in patients with iPD (2). In contrast, levodopa seldom improves lower body bradykinesia in FGD so the postural deficits in these two types of parkinsonism likely have different underlying mechanisms (9, 10).

Anticipatory postural adjustments may also differ in iPD with freezing of gait (FOG) compared with iPD without FOG. Freezing, associated with the impression that the feet are “glued to the floor” can be associated with multiple, large APAs (11, 12). In fact, it has been hypothesized that FOG is due to lack of inhibition of repetitive APAs prior to a step, resulting in “trembling of the knees” (12). FOG eventually affects 80% of people with iPD and is associated with reduced white matter tracks in the right-sided, inhibitory circuitry between the supplementary motor cortex and the subthalamic nucleus of the basal ganglia (6). APAs prior to single-leg stance have not been compared between iPD with and without FOG.

Balance control while standing on a single leg is necessary to accomplish several activities of daily living (e.g., walking, obstacle crossing, and stair climbing) that are required to preserve personal autonomy and a satisfying quality of life (13, 14). Also, the ability to maintain unipedal balance for less than 10 s has been associated with increased fall risk (15–18). The ability to stand on a single limb is therefore an important feature to be assessed in older people and people with parkinsonism. In particular, the One-Leg Stance (OLS) test is a fast and simple tool already adopted for the clinical assessment of balance in the elderly (19, 20) and in subjects with neurological disorders, such as iPD (13, 15–18). Due to its simplicity, the test is also included as an item in more comprehensive clinical scales, such as the Berg Balance Scale (21, 22), the Ataxia Test Battery (23), and the Balance Evaluation System test, both in its complete (BESTest) (24) and short (mini-BESTest) (25) versions. Currently, the only measured outcome in the OLS test is the time the single stance position is held, commonly measured by a stopwatch. However, a previous study of postural steadiness in the OLS test in healthy young and elderly adults (14) underlined the importance of evaluating the anticipatory weight shift toward the stance leg that is a critical balance requirement in daily activities. The OLS task can be divided into two phases: (1) an initial dynamic balance phase, consisting of the postural action of moving the center of mass (CoM) over the forthcoming stance leg, and (2) a following static balance phase in which one leg is lifted while one foot postural orientation is maintained.

Recently, the availability of cost-effective, easy-to-manage, wearable, inertial sensors allows the assessment of motor disorders outside a typical movement analysis laboratory. This wearable technology allows clinicians to easily perform an instrumental evaluation of motor deficits during routine exams. For example, wearable inertial measurement units (IMUs) have been demonstrated to be effective for the assessment of weak APAs that precede step initiation (26–30) and step climbing (28) in iPD. Algorithms to quantify dynamic APAs and static balance associated with OLS are needed.

The aim of this study is to develop and test an instrumented version of the OLS test with wearable inertial sensors that provides objective information of both the dynamic and static phases of the task. The instrumented OLS test was assessed in subjects with iPD, FGD, and elderly healthy adults to characterize: (1) the sensitivity of the method to distinguish differences among groups and (2) the validity of the proposed instrumental indexes for evaluating balance deficits in subjects with different types of parkinsonism.

## Materials and Methods

### Participants

Seventy-one subjects with parkinsonism and a control group of 32 healthy elderly adults (HC) were recruited through the Parkinson’s Center of Oregon at Oregon Health & Science University (OHSU) and VA Portland Health Care System (VAPORHCS). All participants provided informed consent approved by the OHSU or VAPORHCS Institutional Review Boards.

Subjects with parkinsonism were divided into three groups: (a) 25 subjects with idiopathic PD presenting FOG (iPD-FOG), (b) 33 iPD without FOG (iPD-noFOG), and (c) 13 subjects with FGD. Subjects with FGD were included if gait and balance difficulties were the initial symptom of their movement disorder. Clinical features necessary for inclusion were slow short steps, unsteadiness, and difficulty lifting the feet off the floor (shuffling). In addition, wide-based gait, FOG, postural instability, or minor features of parkinsonism (rigidity and tremor) were present in some subjects (optional but supportive for inclusion). For inclusion, clinical characteristics were preferred to radiographic white matter lesion burden. An internationally recognized expert in movement disorders (John G. Nutt) reviewed all the participants with FGD through videos and medical records to confirm inclusion in the FGD group.

Exclusion criteria for subjects with FGD were as follows: idiopathic PD and Parkinson plus syndromes such as progressive supranuclear palsy, multiple system atrophy, corticobasal syndrome, or cerebellar ataxia, Lewy body dementia, and normal pressure hydrocephalus post-shunting. MRI excluded large strokes, masses, cerebellar, and brainstem atrophy or ventricular dilation not related to cortical atrophy (31). Individuals with large, space-occupying lesions on previous imaging or significant pyramidal weakness on exam were also excluded. Other exclusionary criteria were as follows: severe tremor, peripheral neuropathy with proprioceptive deficits, severe peripheral vascular disease, uncorrected vision or vestibular problems, joint disease significantly limiting gait, and inability to tolerate an MRI due to claustrophobia or other medical contraindications.

Subjects were also excluded if they presented: neurological disorders other than iPD or FGD, vestibular disorders, peripheral neuropathy with proprioceptive deficits, musculoskeletal impairments that could affect gait, and inability to stand and walk unassisted. Participants with iPD and FGD were tested in their practical OFF-medication state, after at least 12 h washout from antiparkinson medications.

Subjects with iPD and FGD were clinically rated by a trained examiner on the MDS Motor Section of the Unified Parkinson’s Disease Rating Scale (MDS-UPDRS-III) (32) immediately before the experimental sessions.

Demographic and clinical characteristics of the groups are reported in Table 1.

**Table 1:** Demographic and clinical characteristics of healthy controls (HC), idiopathic Parkinson’s disease without freezing of gait (iPD-noFOG), idiopathic Parkinson’s disease with freezing of gait (iPD-FOG), and frontal gait disorders (FGD) groups.

Group	*N*	Gender (M/F)	Age (years)	Hoehn and Yahr stage (0–5)	UPDRS-III motor section (0–100)
HC	32	15/17	69.4 (7.1)	–	–
iPD-noFOG	33	23/10	67.5 (7.7)	2.1 (0.3)	33.2 (10.7)
iPD-FOG	25	21/4	67.0 (6.5)	2.5 (0.8)	48.2 (14.0)
FGD	13	9/4	73.3 (6.5)	3.2 (0.9)	31.9 (15.9)

*Values are mean (SD) or number*.

### Experimental Protocol

Participants performed the OLS test as part of the mini-BESTest (Item 3). Subjects stood barefoot in an upright posture with feet shoulder-width apart. Their hands were maintained on their hips for the entire duration of the test. In accordance with the general guidelines for the OLS test, participants received the instruction: “Look straight ahead. Keep your hands on your hips. Lift your leg off of the ground behind you without touching or resting your raised leg upon your other standing leg. Stay standing on one leg as long as you can.” The task was performed twice per limb but subjects were not warned in advance which leg to lift so they would not anticipate a weight shift prior to data collection. At the beginning of each repetition, the examiner gave a vocal instruction specifying which leg had to be lifted. Each trial ended after maintaining unipedal balance for 30 s (15) or when the lifted foot touched the ground again.

For concurrent, clinical validity, test duration was also measured with a stopwatch from the movement initiation to the final foot contact. The correspondent clinical task score was assigned, in accordance with the mini-BESTest guidelines, as follows: (0) unable; (1) moderate: *T* < 20 s; (2) normal: *T* ≥ 20 s.

All the remaining 13 items of the mini-BESTest were also performed by subjects and assessed with clinical scores.

**Figure 1:**
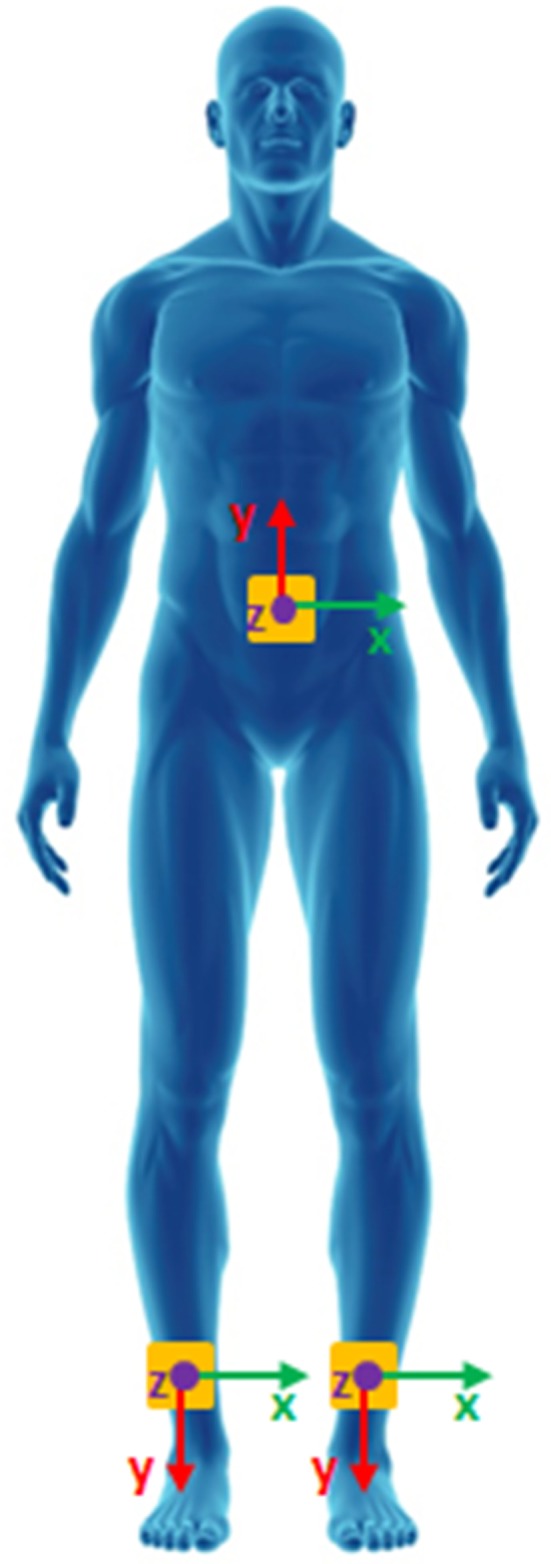
Wearable sensors placement.

Three IMUs (Opals, APDM Inc., Portland, OR, USA), positioned on the posterior trunk at the level of L4–L5, and on the left and right frontal face of the tibias, measured 3D acceleration and 3D angular velocity of the corresponding body segments. The location of the sensors and the orientation of their sensing axes are shown in Figure 1. IMUs were placed directly on the skin and fixed with self-adhering elastic (Coban) bandages. Data were recorded at a sampling frequency of 128 Hz and later downsampled at a frequency of 50 Hz in accordance with previous studies (26, 28–30).

### Data Processing

The acceleration signals recorded from the trunk sensor were transformed to a horizontal–vertical coordinate system (33) and filtered using a fourth-order, zero-phase, low-pass Butterworth filter with a cutoff frequency of 3.5 Hz, as previously proposed in several studies (26, 28–30). The same low-pass filter was adopted for the angular velocities recorded by the sensors on the shanks.

**Figure 2:**
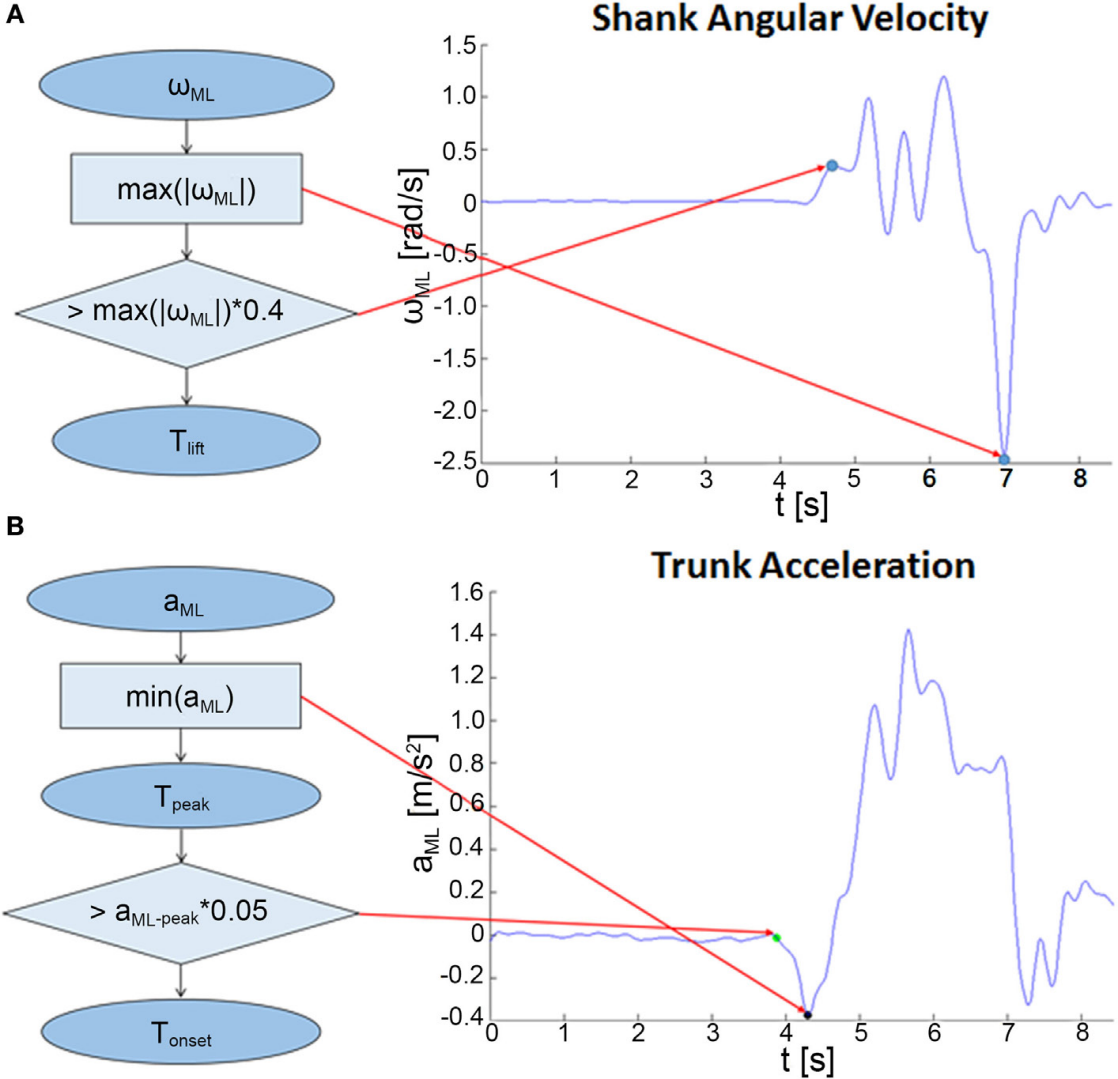
Algorithms for the analysis of the dynamic phase. **(A)** Flowchart describing the procedure for the detection of the beginning of the rising movement of the lifted limb (*T*_lift_). **(B)** Procedure for the identification of the anticipatory postural adjustment onset (*T*_onset_) and the mediolateral (ML)-peak acceleration (*T*_peak_).

For each repetition, the lifted leg was detected automatically on the basis of the highest absolute maximum of the shank angular velocity around the mediolateral (ML) axis (ω_ML_). The initial raising movement of the leg was detected in correspondence of the first instant (*T*_lift_) in which ω_ML_ exceeded a threshold set as 40% of its maximum absolute value, as shown in Figure 2A. The adopted threshold is significantly higher than the value proposed in a previous work on APAs prior to gait initiation and stair climbing (28) to guarantee that the APA phase already ended before *T*_lift_. Thus, starting from the recognized instant *T*_lift_, two different analyses were performed: (1) the assessment of the APAs preceding the leg rising, thus preceding *T*_lift_, and (2) the evaluation of balance during the unipedal stance that follows *T*_lift_.

The former dynamic phase was assessed through the analysis of the trunk ML acceleration, as shown in Figure 2B. The instant corresponding to the maximum absolute peak of the trunk ML acceleration preceding *T*_lift_ was detected (*T*_peak_) and the signal amplitude (ML-peak) was adopted as a descriptive parameter of the APAs (26, 27, 29). Specifically, the ML-peak acceleration was considered representative of the CoM anticipatory spatial behavior because of the demonstrated good correlation with the center of pressure (CoP) displacement measured through a force platform during step initiation (26–29) and stair climbing (28).

The APA onset (*T*_onset_) was then identified as the first instant, starting from the beginning of the recorded signal, in which the trunk ML acceleration exceeded a threshold set as 5% of the extracted ML-peak value.

**Figure 3:**
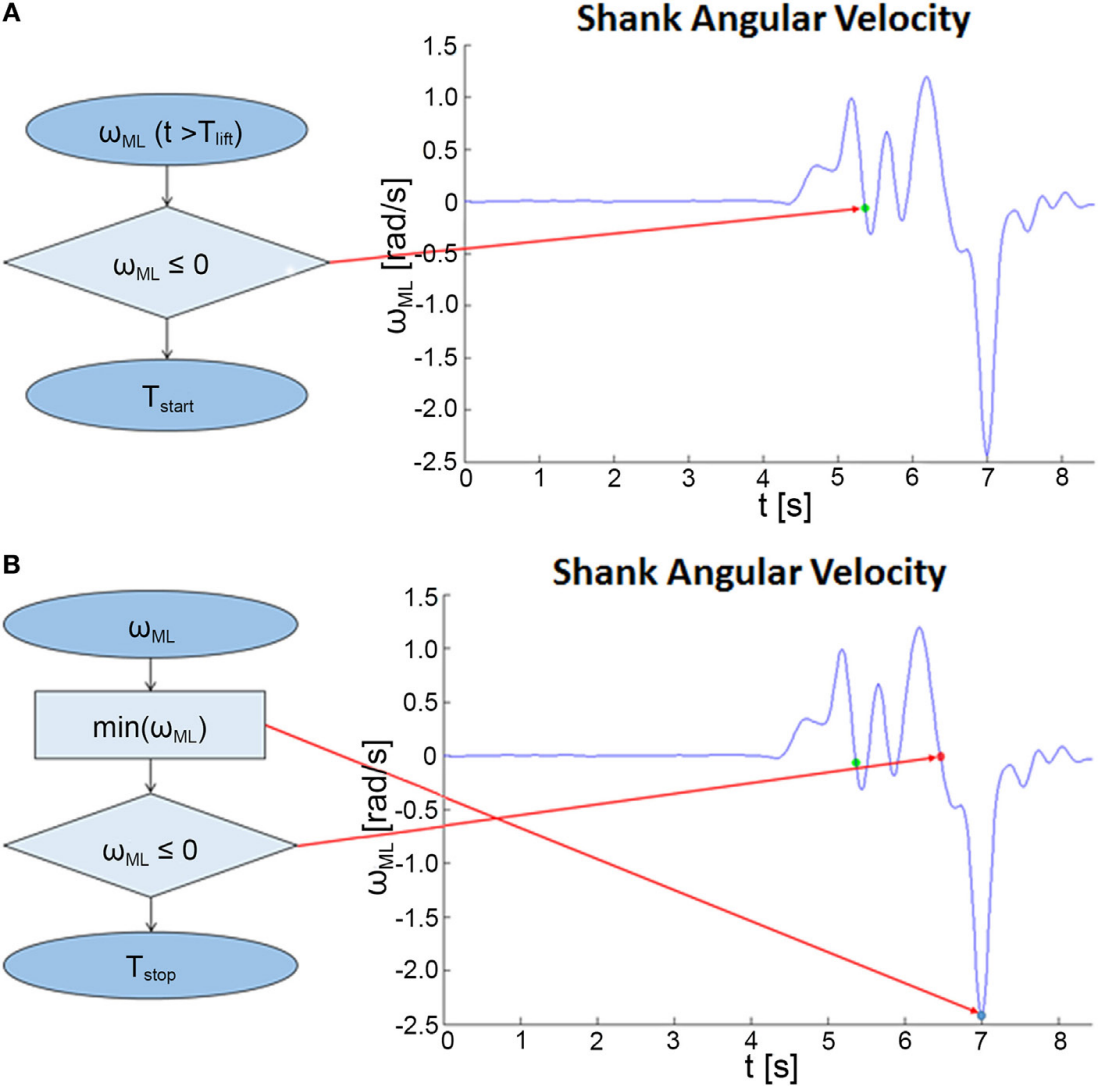
Algorithms for the analysis of the static balance phase. **(A)** Flowchart describing the procedure for the detection of the beginning of the unipedal balance (*T*_start_). **(B)** Procedure for the identification of the end of the unipedal balance (*T*_stop_).

Considering the balance phase that follows *T*_lift_, ω_ML_ was used to detect the initial and final instants of unipedal balance, as reported in Figure 3. The static balance condition while standing on a single limb was reached at the end of the leg lifting, thus at the first instant following the initial rise in which ω_ML_ became negative for the first time (*T*_start_), and ended at the beginning of the final descending movement (detected by the minimum of ω_ML_), hence at the instant in which the shank ω_ML_ became negative for the last time (*T*_stop_).

Following the guidelines for the clinical administration of the OLS test as part of the BESTest (24) and mini-BESTest (25), only the longer trial per leg was considered. In case of equal durations, the selection was performed considering the ML-peak amplitude. Thus, the one presenting the highest amplitude was adopted for subsequent analysis.

The evaluation of body sway during the unipedal balance phase was performed through the analysis of the trunk acceleration. Specifically, the root-mean-square (RMS) values of both the antero-posterior (AP) and ML acceleration were calculated. To take into account the different duration of the balance phase for each subject, RMS values were normalized to the balance duration measured by the sensors (nRMS).

**Figure 4:**
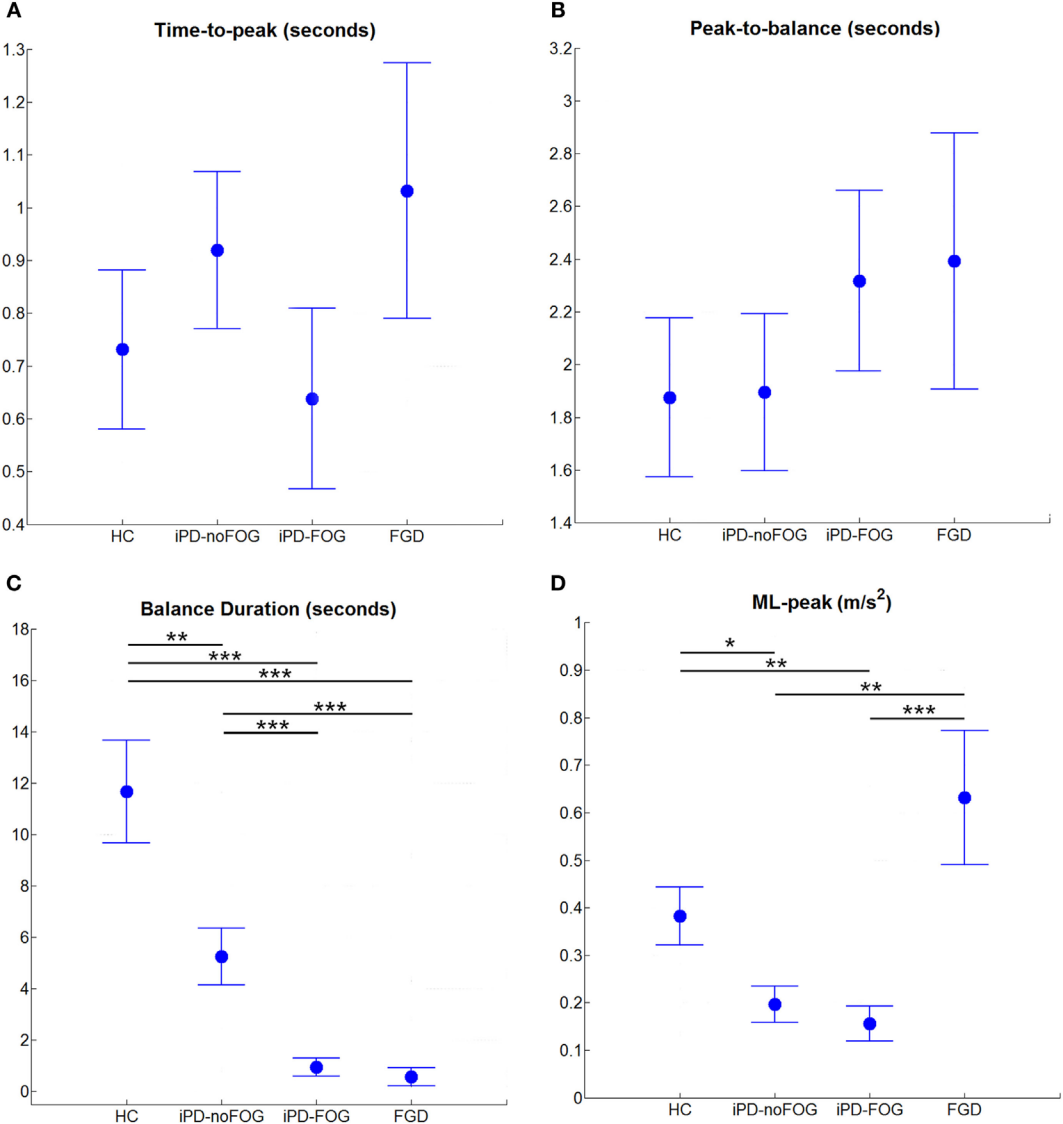
Instrumental parameters extracted from wearable sensors during One-Leg Stance on the most affected side for healthy controls (HC), idiopathic Parkinson’s disease without freezing of gait (iPD-noFOG), idiopathic Parkinson’s disease with freezing of gait (iPD-FOG), and frontal gait disorders (FGD) groups. **(A)** Time-to-peak, **(B)** peak-to-balance, **(C)** balance duration, **(D)** peak of mediolateral trunk acceleration [mediolateral (ML)-peak]. Circles and whiskers represent, respectively, mean and SE adjusted for age through analysis of covariance procedure. *p < 0.05, **p < 0.01, ***p < 0.001 (Bonferroni–Holm *post hoc* comparison).

After the detection algorithm was applied, the following temporal parameters were extracted (Figure 4):

- Time-to-peak: from *T*_onset_ to *T*_peak_;
- Peak-to-balance: from *T*_peak_ to *T*_start_;
- Balance duration: from *T*_start_ to *T*_stop_.

### Statistical Analysis

In accordance with the general guidelines for the OLS test, measures obtained from the most affected side were considered to assess the method sensitivity in discriminating between groups. In addition, the statistical analyses were repeated on the least affected side.

Comparison among the four groups of participants (i.e., HC, iPD-FOG, iPD-noFOG, and FGD) was performed on the parameters extracted through wearable sensors, as well as on the manually measured test durations, and the clinical scores.

Parametric statistical tests were used for the analysis of data extracted through wearable sensors or stopwatch. Data normality and homogeneity of variances were tested with Kolmogorov–Smirnov test and Levene’s test, respectively. In case of variables that did not meet the above mentioned assumptions, the statistical analysis was performed on data transformed with Box–Cox transformation (34).

To reduce the effect of age which showed an almost significant difference between FGD subjects and the two groups of iPD participants (*p* = 0.07 and 0.06, respectively), between-group comparisons were assessed by analysis of covariance (ANCOVA) with one between-group factor (group: HC, iPD-FOG, iPD-noFOG, and FGD) and the factor “age” as covariate. In case of significant difference (*p* < 0.05), separate *post hoc* comparisons were performed using Bonferroni–Holm procedure.

The clinical scores of the OLS test as proposed in the mini-BESTest, the Anticipatory Subscore, and the total mini-BESTest score were analyzed through non-parametric methods due to their discrete nature. Thus, differences among groups were assessed using the Kruskal–Wallis Rank Sum Test and Bonferroni–Holm *post hoc* procedure. The same procedure was adopted to investigate differences in the MDS-UPDRS Part 3—Motor Subscore among subjects with iPD-noFOG, iPD-FOG, and FGD.

Considering the discrete nature of the mini-BESTest clinical score, the Spearman’s rank correlation coefficient (ρ) was used to investigate the associations between the balance duration measured through wearable sensors and the clinical score. Pearson’s correlation coefficient (*r*) was used to investigate the associations between the parameters extracted by wearable sensors and the test duration measured through a stopwatch. In both cases, the strength of all correlations was interpreted as follows: trivial (*r* < 0.1), small (0.1 < *r* < 0.3), moderate (0.3 < *r* < 0.4), strong (0.5 < *r* < 0.7), very strong (0.7 < *r* < 0.9), and perfect (*r* = 1.0) (35). Bland–Altman analysis was also carried out to investigate the relationship between balance duration measured through wearable sensors and the stopwatch (36).

The level of significance was set at 0.05 for all the conducted analyses. All the analyses were performed using R (R Foundation for Statistical Computing, Vienna, Austria).

## Results

All the participants completed the test. However, considering the execution of the test on the most affected side, 13 subjects with parkinsonism (1 iPD-noFOG, 8 iPD-FOG, and 4 FGD) were not able to lift their leg, while considering the least affected side only 3 persons (1 iPD-noFOG, 1 iPD-FOG, and 1 FGD) could not get the foot off the ground. Due to the impossibility to maintain unipedal balance, subjects who did not get their foot off the ground were excluded from the assessment of the body sway during the balance phase by lower trunk acceleration data.

### Clinical Scores of OLS Differentiate Parkinsonism from Healthy Controls

Results are reported in Table 2. Specifically, statistically significant differences among groups were found for the OLS task of the mini-BESTest, the Anticipatory Postural Subscore, and the mini-BESTest total score (*p* < 0.001). Differences in the OLS task score were also found between healthy controls (mean ± SD: 1.6 ± 0.6) and iPD-noFOG (1.2 ± 0.6, *p* = 0.01), iPD-FOG (0.9 ± 0.6, *p* < 0.001), and FGD (0.5 ± 0.6, *p* < 0.01). Furthermore, FGD presented significant lower score when compared to iPD-noFOG (*p* = 0.002) and iPD-FOG (*p* = 0.04), but no differences were found between iPD groups (*p* = 0.15). Analyses of the Anticipatory Subscore showed differences between all the groups, but not between iPD-FOG and FGD subjects (iPD-FOG: 3.2 ± 1.4, FGD: 2.6 ± 1.3, *p* = 0.25). In particular, HC (5.0 ± 1.2) had higher score than iPD-noFOG (4.3 ± 1.3, *p* = 0.03), iPD-FOG, and FGD (*p* < 0.001). Similarly, iPD-noFOG reported higher score than iPD-FOG (*p* = 0.01) and FGD (*p* < 0.001). Finally, the mini-BESTest total score demonstrated the highest sensitivity by discriminating all the four considered groups of participants (*p* < 0.02 for all the comparisons).

**Table 2:** Comparison of the clinical measures among healthy controls (HC), idiopathic Parkinson’s disease without freezing of gait (iPD-noFOG), idiopathic Parkinson’s disease with freezing of gait (iPD-FOG), and frontal gait disorders (FGD).

	HC	iPD-noFOG	iPD-FOG	FGD	*p*-Value
Mini-BESTest One-Leg Stance Task score (0–2)	1.6 ± 0.6	1.2 ± 0.6	0.9 ± 0.6	0.5 ± 0.6	<0.001
Mini-BESTest Anticipatory Subscore (0–6)	5.0 ± 1.2	4.3 ± 1.3	3.2 ± 1.4	2.6 ± 1.3	<0.001
Mini-BESTest Total Score (0–28)	24.2 ± 2.4	21.3 ± 4.0	16.6 ± 5.7	11.9 ± 5.3	<0.001

*Values are mean ± SD. Higher scores indicate better performance. Reported p-values refer to Kruskal–Wallis Rank Sum Test*.

### Specific Characteristics of the Instrumented OLS Differentiate between Parkinsonism and Healthy Controls

Results related to the most affected side are reported in Figure 4. Regarding the anticipatory phase, the peak of ML trunk acceleration (ML-peak) revealed a statistically significant difference among groups [*F*(3, 98) = 7.94, *p* < 0.001]. As shown in Figure 4D, the ML-peak was significantly lower in the two iPD groups (iPD-noFOG: *p* = 0.027; iPD-FOG: *p* = 0.007) compared to healthy controls, and surprisingly compared to FGD (iPD-noFOG: *p* = 0.002; iPD-FOG: *p* < 0.001). Also surprisingly, the ML-peak was similar in subjects with FGD compared to healthy controls (*p* = 0.151). Considering the body sway during the unipedal balance phase (Table 3), differences among groups in normalized root-mean-square acceleration (nRMS) were found in both the AP [AP-nRMS, *F*(3, 85) = 4.83, *p* = 0.004] and ML [ML-nRMS, *F*(3, 85) = 7.49, *p* < 0.001] directions. *Post hoc* analysis showed that healthy controls were characterized by significant lower nRMS values, thus lower body sway, in both the AP (iPD-noFOG: *p* = 0.040; iPD-FOG: *p* = 0.036; FGD: *p* = 0.018) and ML (iPD-noFOG: *p* = 0.002; iPD-FOG: *p* = 0.004; FGD: *p* = 0.005) directions. No difference among the three groups of subjects with parkinsonism was found.

**Table 3:** Normalized root mean square of the lower trunk acceleration during unipedal balance on the most and least affected sides for healthy controls (HC), idiopathic Parkinson’s disease without freezing of gait (iPD-noFOG), idiopathic Parkinson’s disease with freezing of gait (iPD-FOG), and frontal gait disorders (FGD) groups.

		HC	iPD-noFOG	iPD-FOG	FGD	*p*-Value
Most affected	Antero-posterior (AP)-nRMS (m/s^3^)	0.027 ± 0.007	0.071 ± 0.018	0.085 ± 0.030	0.141 ± 0.071	0.004*
	Mediolateral (ML)-nRMS (m/s^3^)	0.045 ± 0.010	0.152 ± 0.036	0.172 ± 0.055	0.253 ± 0.116	<0.001*
Least affected	AP-nRMS (m/s^3^)	0.012 ± 0.002	0.017 ± 0.004	0.023 ± 0.006	0.029 ± 0.011	0.067
	ML-nRMS (m/s^3^)	0.026 ± 0.005	0.035 ± 0.007	0.054 ± 0.014	0.076 ± 0.029able 5	<0.028*

Values are mean ± SE adjusted for age through analysis of covariance procedure. Significant differences (*p*-value < 0.05) are marked with *.

Regarding temporal aspects, a statistically significant difference among groups emerged only for balance duration [*F*(3, 98) = 24.07, *p* < 0.001], while no significant differences were found for the time-to-peak [*F*(3, 98) = 0.89, *p* = 0.448] and peak-to-balance [*F*(3, 98) = 0.58, *p* = 0.628] (Figures 4A,B). Further *post hoc* analysis (Figure 4C) showed that healthy control subjects were able to maintain balance longer than subjects with iPD-noFOG (*p* = 0.007), iPD-FOG (*p* < 0.001), and FGD (*p* < 0.001). Furthermore, iPD-noFOG subjects stayed in unipedal stance longer than iPD-FOG (*p* < 0.001) and FGD (*p* < 0.001), while no difference was found between iPD-FOG and FGD (*p* = 0.489). These results were confirmed by ANCOVA analysis conducted on the test duration measured through stopwatch [*F*(3, 98) = 21.54, *p* < 0.001]. Healthy elderly controls presented longer, thus better, time (mean ± SE: 14.6 ± 1.5 s) than iPD-noFOG (7.7 ± 1.1 s, *p* < 0.001), iPD-FOG (3.8 ± 0.9 s, *p* < 0.001), and FGD (1.8 ± 0.8 s, *p* < 0.001). Furthermore, iPD-noFOG subjects presented better performances than iPD-FOG (*p* = 0.013) and FGD (*p* < 0.001), while no difference was found between iPD-FOG and FGD (*p* = 0.117).

Data from the least affected side are summarized in Tables 3 and 4. Regarding the anticipatory phase, no differences were found among groups for the peak of ML trunk acceleration [*F*(3, 98) = 1.82, *p* = 0.148], in contrast with the analysis of the most affected side (Table 4). Analysis of the normalized body sway during unipedal balance (Table 3) showed a significant difference among groups only in the ML direction [*F*(3, 95) = 3.16, *p* = 0.028]. However, after *post hoc* analysis, only a tendency toward significance was found between HC and FGD groups (*p* = 0.066).

**Table 4:** Instrumental parameters extracted from wearable sensors during One-Leg Stance on the least affected side for healthy controls (HC), idiopathic Parkinson’s disease without freezing of gait (iPD-noFOG), idiopathic Parkinson’s disease with freezing of gait (iPD-FOG), and frontal gait disorders (FGD) groups.

	HC	iPD-noFOG	iPD-FOG	FGD
Balance duration (s)	19.81 ± 1.90	13.37 ± 1.54	6.56 ± 1.24	6.40 ± 1.74
Time-to-peak (s)	0.39 ± 0.08	0.42 ± 0.09	0.39 ± 0.10	0.44 ± 0.15
Peak-to-balance (s)	1.31 ± 0.16	1.22 ± 0.15	1.39 ± 0.19	0.92 ± 0.19
ML-peak (m/s^2^)	0.34 ± 0.05	0.25 ± 0.04	0.21 ± 0.04	0.33 ± 0.07

*Values are mean ± SE adjusted for age through analysis of covariance procedure*.

Results related to the temporal aspects (Table 4) confirmed those obtained from the most affected side, showing a significant difference among groups for balance duration [*F*(3, 98) = 14.74, *p* < 0.001], which was significantly prolonged in control subjects compared to iPD with and without FOG (*p* = 0.019 and *p* < 0.001, respectively) and subjects with FGD (*p* < 0.001) and in iPD-noFOG compared to iPD-FOG (*p* = 0.004) and FGD (*p* = 0.020). No differences among groups were found for the other temporal parameters [time-to-peak: *F*(3, 98) = 0.05, *p* = 0.986; peak-to-balance: *F*(3, 98) = 1.02, *p* = 0.389], in accordance with the results from the most affected side. The statistical analysis performed on the test duration measured with a stopwatch confirmed differences among groups [*F*(3, 98) = 13.26, *p* < 0.001]. *Post hoc* analysis highlighted that healthy controls presented longer unipedal standing (21.8 ± 1.5 s) than iPD-noFOG (15.3 ± 1.5 s, *p* = 0.010), iPD-FOG (10.7 ± 1.7, *p* < 0.001), and FGD (6.9 ± 2.3, *p* < 0.001). iPD-noFOG showed longer time than FGD (*p* = 0.011), but no differences were found between iPD-FOG and iPD-noFOG (*p* = 0.08) and between iPD-FOG and FGD (*p* = 0.193).

### Correlation between Clinical and Instrumented Features of the OLS Test

**Figure 5:**
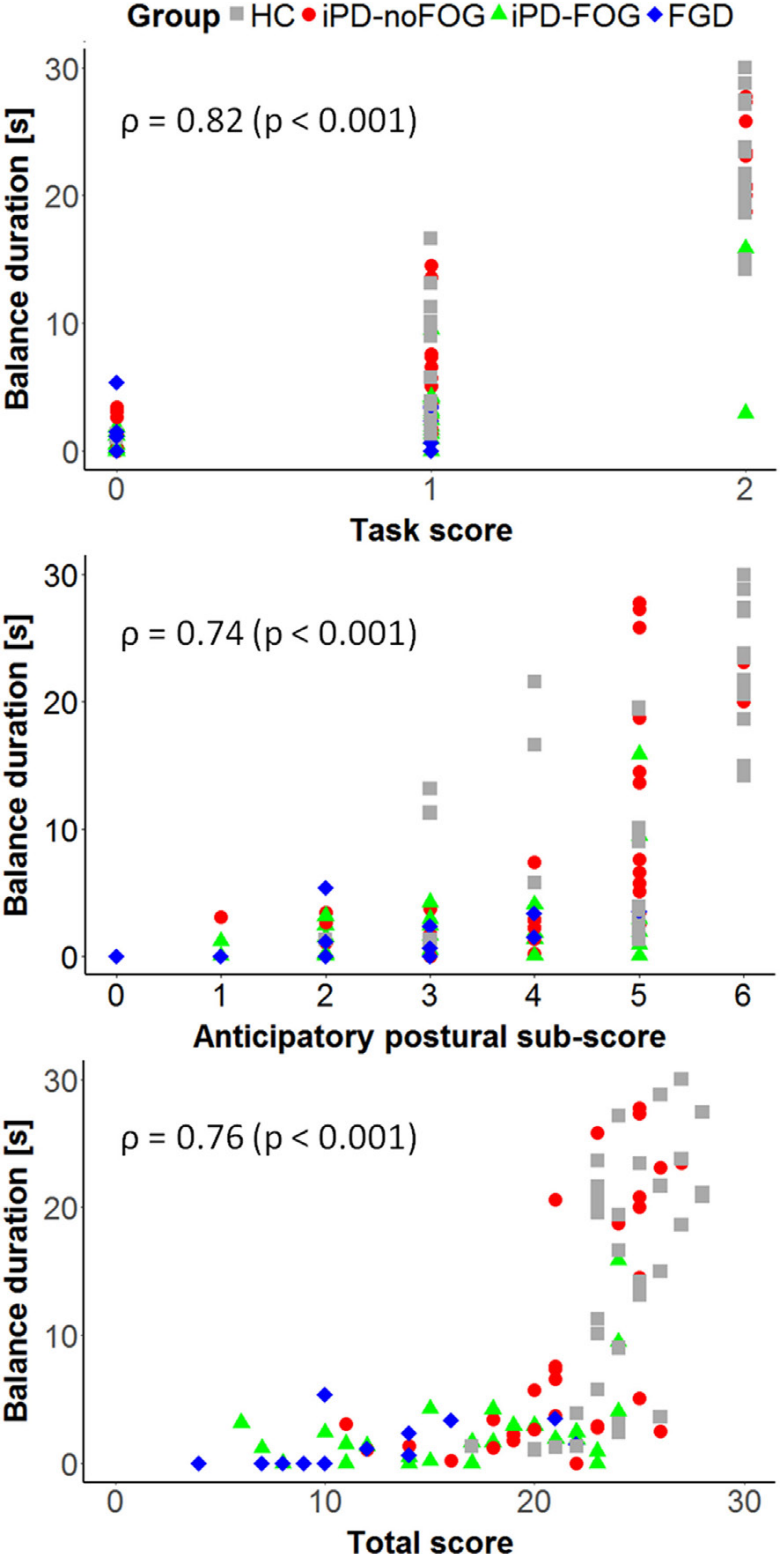
Correlation between the balance duration on the most affected leg measured through the wearable sensors and the mini-BESTest task score (top), Anticipatory Subscore (center), and total score (bottom). Spearman’s rank correlation coefficient (ρ) and the correspondent *p*-values are reported.

**Figure 6:**
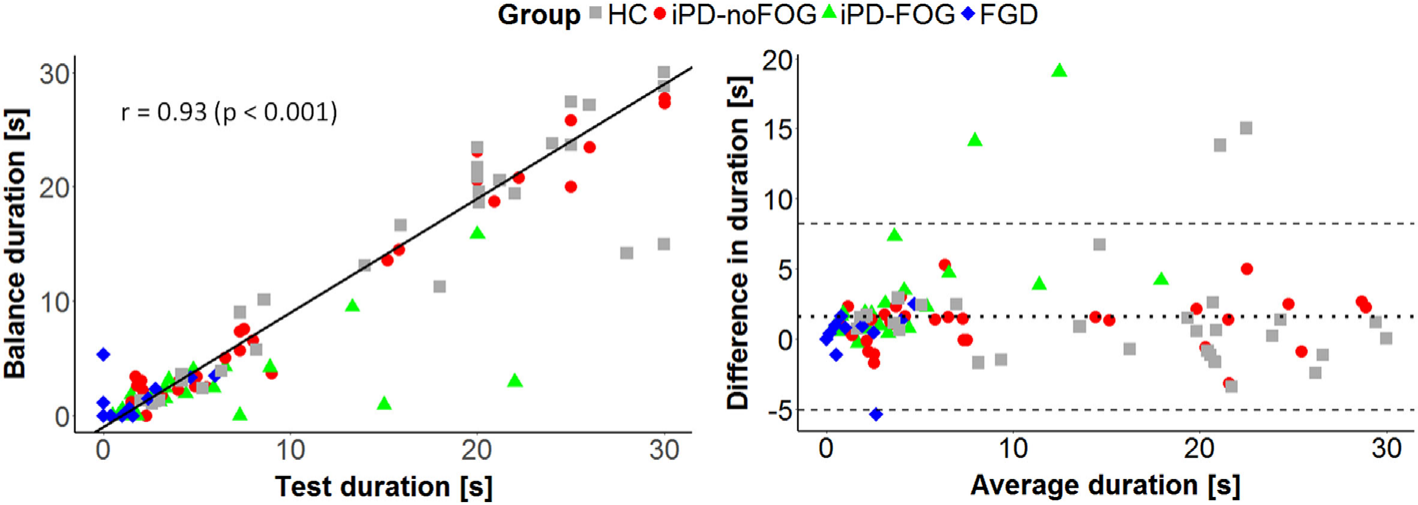
Comparison between balance duration measured through wearable sensors and task duration measured by stopwatch during the execution of the One-Leg Stance test on the most affected side. On the left, linear correlation between the two variables. Pearson’s correlation coefficient (*r*) and the correspondent *p*-value are reported. On the right, Bland–Altman plot. The central dotted lines represent the mean difference between the two measures, while the upper and lower lines represent the limits of agreement.

Figures 5 and 6 summarize the results of the correlation analysis between clinical and instrumental parameters related to the most affected side.

The balance duration measured by wearable sensors presented a strong correlation with the mini-BESTest OLS task score (ρ = 0.82, *p* < 0.001), the mini-BESTest Anticipatory Subscore (ρ = 0.74, *p* < 0.001) and the mini-BESTest total score (ρ = 0.76, *p* < 0.001) (see Figure 5), while no significant correlation was found between balance duration and the MDS-UPDRS-III Motor Subscore (ρ = −0.15, *p* = 0.219). In contrast, ML-peak acceleration presented only a moderate correlation with the MDS-UPDRS-III Motor Subscore (ρ = −0.33, *p* = 0.012), and no significant correlation was found with the other clinical measures. As shown in Figure 6 (left panel), a very strong correlation (*r* = 0.93, *p* < 0.001) emerged between the task duration measured with a stopwatch and the balance duration computed from wearable sensors. Besides, Bland–Altman analysis (Figure 6, right panel) showed no obvious relation between the difference and the mean value of the balance duration measured by wearable sensors and test duration measured by stopwatch. Moreover, the mean value of the difference in duration between the two measures $\left(\bar{\Delta t}=1.6\text{s}\right)$ can be ascribed to the difference in the measured intervals. Indeed, the instrumental measure of the unipedal balance phase excludes the initial rise and final fall of the leg, while these transient movements are considered in the measure performed with stopwatch. Taking into account that the two methods do not evaluate the same exact intervals, the collected results suggest an equal agreement between the proposed and the traditional methods through the entire range of measurements.

The above results were confirmed by data related to the least affected side, which also showed a very strong correlation between the balance duration measured by wearable sensors and the mini-BESTest task score (ρ = 0.71, *p* < 0.001), the Anticipatory Subscore (ρ = 0.69, *p* < 0.001), and the total score (ρ = 0.76, *p* < 0.001). Moreover, the lack of significant correlation between the ML-peak acceleration and the clinical mini-BESTest scores was found also for the least affected side. In addition, no correlation with the MDS-UPDRS-III Motor Subscore was found neither for the balance duration nor for the ML-peak acceleration.

## Discussion

This study developed and tested algorithms for an instrumented version of the OLS test based on wearable inertial sensors with healthy people, subjects with idiopathic PD, with and without FOG, and subjects with FGD. Our findings support the validity of the proposed method for assessing the OLS test and its sensitivity in distinguishing among the tested groups. Specifically, the objective characterization of dynamic ML acceleration of the trunk during the APAs that precede the unipedal static balance phase represents an improvement of the discriminatory ability of the OLS test compared to most clinical tests of OLS. The instrumented OLS test discriminated between healthy older people and people with parkinsonism and was sensitive to all the groups with parkinsonism. Only the complete mini-BESTest presented similar sensitivity to distinguish performance among group balance performance.

To the best of our knowledge, this is the first study in which wearable sensors were used for assessing both the initial dynamic (i.e., APAs and leg lifting) and the subsequent static balance phases of the OLS task. The importance of the evaluation of the two phases has been already reported in laboratory studies (14) as necessary for the correct comprehension of the weight-shifting mechanisms, but the use of IMUs for the objective observation of the phases was never investigated. Previously, the assessment of the dynamic and static phases of the task was performed with a force plate (14), that is, generally confined to motion analysis laboratories. Two previous studies developed a wearable sensor-based version of the OLS test for assessing balance deficits and risk of falling. In one study, the test was instrumented by using sensorized insoles for the estimation of CoP parameters (37), while in a second study a trunk-worn smartphone was used to estimate trunk displacements during task execution without considering the APA phase and balance phase separately (38). In addition, the present work is the first effort to adopt an instrumented version of the OLS test to discriminate subjects with idiopathic PD, with and without FOG, and FGD.

Four temporal (time-to-peak, time-to-balance, peak-to-balance, and balance duration) and three accelerometric (ML-peak acceleration and the normalized RMS of the lower trunk acceleration in the AP and ML directions) features were automatically extracted. The acceleration of the lower trunk has already shown to be appropriate for describing the behavior of the CoM during APAs preceding intentional movements in healthy elderly and young adults and subjects with iPD (26–29). In all those studies, the ML component of the acceleration had a good correlation with the CoP displacement measured through a force platform, while the acceleration in the AP direction had weaker correlations (26, 29) or no correlation at all (28). Therefore, on the basis of these results, only the ML-peak acceleration was adopted in our study as a spatial parameter to characterize APAs. The RMS acceleration of the lower trunk in the AP and ML directions was adopted to assess the postural sway while standing on a single leg. Indeed, this parameter has been already addressed as a valid and reliable measure to characterize postural control in PD (39). In this study, to take into account the ability in maintaining unipedal balance of subjects with different clinical conditions, the extracted measures were normalized to the corresponding balance duration, automatically extracted by the sensors. Finally, the four temporal features were intended to characterize the different subcomponents of the dynamic phase and the static balance phase. In particular, Mancini et al. (26) reported that the time-to-peak trunk acceleration correlated with both the amplitude of the CoM acceleration and the CoP displacement in APAs preceding gait initiation.

A significant difference in ML-peak acceleration was found between healthy elderly and iPD subjects, with and without FOG. This result agrees with the literature, as people with iPD also show hypometric APAs in other motor tasks, such as gait initiation (26–30) and stair climbing (28). The reduced initial shift of the CoM prior to step initiation has been associated with start hesitation and akinetic FOG (12, 40). Thus, it has been suggested that the typical alteration in postural control during the initial dynamic phase may determine an insufficient recruitment and an under-scaled muscle force (13) that can result in balance difficulties in the subsequent static phase (13, 14, 19).

Subjects with iPD and FOG showed a reduced ability to maintain balance on a single leg, resulting in a significant shorter balance duration compared to subjects with iPD without FOG. This result might either reflect the higher level of motor impairment in this cohort of iPD presenting with FOG, as confirmed by a significantly worse, MDS-UPDRS Part 3—Motor Subscore, or be the result of differentially impaired control of balance in subjects experiencing freezing (41). No other differences were found between iPD-FOG and iPD-noFOG in the other objective measures.

Subjects with FGD showed ML-peak acceleration significantly higher than subjects with iPD and comparable with healthy controls. This very interesting result of normal APA size seems to be in contrast with the FGD subjects’ short one-legged balance duration as well as their worst, mini-BESTest total score, worst Anticipatory Subscore and worst OLS subscore. However, FGD subject may not correctly scale the consequent weight shift to maintain the CoM projection inside the base of support, thus resulting in short lifting attempt, immediately followed by ground foot contact. These results are similar to those reported in a previous study on gait initiation by Elble et al. (42). In that case, subjects with lower body parkinsonism (FGD) showed initial postural shifts on a force plate qualitatively similar to those of comparable-aged, healthy controls, with no significant differences in the amplitude of the ML CoP displacement. However, due to a significant reduction in the moment of force measured by a force plate in the AP direction, people with FGD had abnormal postural shifts followed frequently by one or more aborted attempts at lifting the foot. Since patients with FGD do not benefit from dopamine replacement therapy, it is likely their parkinsonian balance and gait problems, and inability to stand on one foot, stem from deficits in different neural circuits than those with iPD (9).

Significant differences were found in duration of the one foot balance phase between healthy elderly subjects and the groups with parkinsonism, as well as between the iPD with FOG versus the non-FOG and FGD group, respectively. However, no significant differences were found between iPD-FOG and FGD group performance. Reduced one foot balance times are generally associated with poor balance capabilities and higher fall risk (15–18, 43), so the differences in balance duration between groups seem to correctly reflect different levels of balance abnormality, confirmed by the clinical mini-BESTest assessments. In particular, the lack of a significant difference between iPD-FOG and FGD subjects in the mini-BESTest’s Anticipatory Subscore suggests that those subjects presented similar anticipatory balance deficits, even though FGD also had worse balance in other domains than the iPD-FOG group, as showed by the mini-BESTest’s total score. No other differences in temporal parameters were found among groups, in line with previous studies demonstrating that time-to-peak did not discriminate between healthy older adults and subjects with iPD during gait initiation (26, 29).

The higher values of the nRMS showed by subjects with parkinsonism while standing on their most affected side compared to healthy controls correctly reflect the poor control of posture typical of iPD (44) and the spectrum of locomotor impairment comprising postural instability that are associated with frontal lobe pathology (42).

The very strong correlation found between the balance duration extracted through wearable sensors and the test duration measured by a stopwatch, and the strong correlation with the mini-BESTest one foot standing task score, Anticipatory subscore and total score support the validity of our parameters for assessing the OLS test. This fact is enforced by Bland–Altman analysis that shows no obvious relationship between the difference and the mean value of the balance duration measured by wearable sensors and test duration measured by stopwatch. By contrast, no significant correlation was found between balance duration and disease severity, as measured by the MDS-UPDRS-III Motor Subscore. This result could be ascribed to the fact that MDS-UPDRS-III scale contains not only subscores specific to balance stability and gait, typically impaired in subjects with both iPD and FGD, but also components related to tremor, rigidity and arm bradykinesia, which are typical of iPD but that are not usually present in people with FGD (6).

The proposed, instrumented method of assessing one foot standing demonstrated a higher sensitivity compared to the generally adopted stopwatch approach. In fact, the analysis of both the initial dynamic phase (by ML-peak acceleration) and of the subsequent static balance phase (by balance duration) allowed us to correctly differentiate between healthy elderly and subjects with parkinsonism, and to discriminate among groups with different types of parkinsonisms and disease stages. Notably, the ability to characterize the initial APAs prior to leg lift represents a novel, clinically valuable evaluation of unipedal balance.

In accordance with the general guidelines for the OLS test as presented in the mini-BESTest, the analysis was performed considering the best performance from the most affected side. Further investigation conducted by repeating the data analysis on the least affected side confirmed lower duration of the balance phase for subjects with iPD or FGD compared to healthy controls and for subjects with iPD-FOG and FGD compared to iPD-noFOG. However, no significant differences were found among groups in the anticipatory phase. This result suggests the possibility that the ability to generate the anticipatory adjustments needed for the OLS test may be preserved in people with parkinsonism. Considering that APAs are known to be asymmetric in healthy subjects (45–47) and even more in subjects with iPD (48), this result supports the practice of performing the test on the most affected side to exacerbate differences in balance control.

Some limitations are present in this study. The first limitation is represented by the small number of subjects with FGD involved in the study. The limited size of this cohort and the lack of previous studies on the APAs in FGD suggest caution in data interpretation. Second, the adoption of ML-peak acceleration as a discriminatory parameter of APAs is based on previous studies already published, that showed a good correlation between the acceleration signals measured by an inertial sensor placed on the lower trunk and the CoP displacement in quiet standing and prior to step initiation, however no similar assessment has been conducted yet for FGD subjects. These considerations suggest that further studies are needed to validate the proposed method on a wider cohort of subjects with FGD. Moreover, a comparison between data from inertial sensors and a force plate (considered as gold standard) should be performed to allow an improvement of the algorithms for the detection of heel-off and toe-off instants, permitting a further assessment of the imbalance and unloading phases, separately (28, 30).

Even though caution is needed, based on previous studies where similar algorithms were used, it is opinion of the authors that the proposed method appears to be robust enough for subjects with parkinsonism. The adoption of cost-effective, wearable sensors allowed us to enhance the sensitivity of the OLS test, without introducing any further complexity. This represents a potentially useful instrument for the fast assessment of balance deficits in clinical settings.

## Ethics Statement

This study was carried out in accordance with the recommendations of Ethics Committee of the Oregon Health & Science University and of the VA Portland Healthcare System with written informed consent from all subjects. All subjects gave written informed consent in accordance with the Declaration of Helsinki. The protocol was approved by the Ethics Committee of the Oregon Health & Science University and of the VA Portland Healthcare System.

## Author Contributions

FH and MM designed the work. MM and GB performed data acquisition. GB implemented algorithms for data processing and drafted the manuscript. GB and IC analyzed data. FH, LC, MF, and JN contributed to the interpretation of the data. FH, MM, IC, LC, MF, and JN participated in the critical revision process. All the authors approved the manuscript.

## Conflict of Interest Statement

FH had a significant financial interest in APDM, a company that may have a commercial interest in the results of this research and technology. This potential institutional and individual conflict has been reviewed and managed by OHSU and the Portland VA.
